# Fear of Being Judged: A Phenomenological Hermeneutical Study of Encountering Ambulance Staff When Living in Stigmatized Neighbourhoods

**DOI:** 10.1111/scs.70311

**Published:** 2026-09-01

**Authors:** Sara Björklund, Peter Hagell, Petra Lilja Hagell, Mats Holmberg

**Affiliations:** ^1^ The PRO‐CARE Group, Faculty of Health Sciences Kristianstad University Kristianstad Sweden; ^2^ Ambulance Service Department Region Blekinge Karlshamn Sweden; ^3^ Center of Interprofessional Collaboration Within Emergency Care Linnaeus University Växjö Sweden; ^4^ Faculty of Health and Life Sciences Linnaeus University Växjö Sweden; ^5^ Ambulance Service Department Region Sörmland Katrineholm Sweden; ^6^ Center for Clinical Research Sörmland Uppsala University Eskilstuna Sweden

**Keywords:** ambulance, caring encounters, minority stress, nurse–patient relations, person‐centeredness, stigmatization, street‐level bureaucracy

## Abstract

**Aims and Objectives:**

To elucidate meanings of encountering ambulance staff when living in stigmatized neighbourhoods.

**Methodological Design and Justification:**

The study was conducted using a phenomenological hermeneutical approach based on narrative interviews.

**Ethical Issues and Approval:**

The study was approved by the Swedish Ethical Review Authority and conducted in accordance with the Declaration of Helsinki.

**Research Methods:**

A phenomenological hermeneutical analysis was conducted. Interviews were performed with 18 persons living in stigmatized neighbourhoods who had lived experiences of encountering ambulance staff, either as relatives or as patients.

**Results:**

These encounters carry meanings of being dependent on how the staff act in situations marked by fear, involving fear for one's own or a relative's health, or fear of being judged based on ethnicity, culture, or place of residence.

**Study Limitations:**

The participants shared similar demographic backgrounds, and recruitment through gatekeepers may have constrained the range of perspectives. As an interpretive method, the findings represent one possible understanding, and other interpretations may emerge from different backgrounds or theoretical lenses.

**Conclusions:**

Encounters with ambulance staff carried meanings of fear of being judged and of dependence, while ambulance staff's interpersonal actions could either reinforce or alleviate these meanings.

## 1 Introduction

Residential areas can be subject to various forms of institutional classification through policy categorizations, administrative practices and public representations [1]. Such classifications may influence both the symbolic and material conditions of residential areas by shaping public perceptions, opportunities, access to resources and patterns of investment and disinvestment [2]. When residential areas become symbolically associated with social problems such as poverty and crime, they may become subject to processes of neighbourhood stigmatization, whereby negative meanings become attached to both places and the persons living within them [3]. Such processes have been associated with reduced opportunities, social exclusion and the reproduction of inequalities affecting both residents and local communities [1, 2].

In Sweden, the Police Authority has defined and publicly published a list of neighbourhoods described as having socioeconomic deprivation and criminality that impacts the local community [4]. Although intended to support crime prevention, this list has drawn broader attention and may reinforce negative understandings, thereby contributing to neighbourhood stigmatization [5]. As many stigmatized neighbourhoods are characterized by high proportions of residents with migration backgrounds and socioeconomic disadvantage, stigma becomes intertwined with ethnicity, marginalization and structural inequalities [3]. This can result in cumulative disadvantages, where residents are affected not only by place‐based stigma but also by broader processes of social and institutional exclusion, including within health care services [3, 6].

In this context, access to ambulance services (AS) may be particularly important, as they are often contacted in urgent health‐related situations and are responsible for assessing needs and providing care on site [7]. Previous research suggests that living in a stigmatized neighbourhood may not only constrain access to the AS but also increase the risk of being understood by ambulance staff through stereotyping based on ethnicity, culture, or place of residence, which may affect the care provided [6, 8, 9]. Taken together, this indicates that neighbourhood stigmatization may shape AS health care encounters and potentially influence the care provided. However, existing knowledge remains limited regarding how such encounters are experienced by patients and relatives in need of AS provided care. Therefore, this study aimed to elucidate meanings of encountering ambulance staff when living in stigmatized neighbourhoods.

## 2 Methods

This qualitative study is based on a phenomenological hermeneutical approach devised by Lindseth and Norberg [10], which aims to elucidate the meanings of a phenomenon. The approach draws on Husserl's phenomenology as an ontology [11], in which a phenomenon is understood as that which reveals itself, and its meaning can be disclosed through narratives of lived experience. To access deeper meanings rather than surface characteristics, the approach is epistemologically informed by Ricoeur's hermeneutic philosophy [12]. This approach is operationalized through an interpretative process that moves between Ricoeur's hermeneutic concepts of explanation and understanding, where explanation aims to describe the phenomenon and its parts, while understanding interprets its subjective and contextual significance [12]. This guides the analysis as a dynamic movement between the whole and its parts, whereby meanings are progressively disclosed through iterative engagement with the text. This movement is reflected in the progression from a naive understanding of the text as a whole, through structural analysis focusing on its parts, to a comprehensive understanding in which the interpreted meanings are synthesized [10] (see Section 2.4).

### 2.1 Setting

The study was conducted in a Swedish neighbourhood included in a list of areas defined by the Swedish Police Authority [4]. The list contains 65 neighbourhoods with a total population of about 500,000 persons (5% of the Swedish population) [4]. These neighbourhoods vary in population size (1300 to 35,000 persons) as well as characteristics. In comparison to the rest of Sweden, they have a younger population with a large number of persons born in another country, as well as a higher risk of socioeconomic deprivation [13].

### 2.2 Participants and Recruitment Procedure

A purposive sampling strategy using gatekeepers and snowball sampling was employed, as these approaches are considered suitable for accessing populations that are less likely to be reached through standardized recruitment methods [14]. Recruitment was planned in collaboration with reference groups from local non‐profit organizations, who helped identify potential gatekeepers. The first author contacted the gatekeepers, informed them about the study and invited them to support participant recruitment. Gatekeepers who consented were engaged in community activities such as homework support, labour exchange, religious leadership and local politics. Gatekeepers informed potential participants about the study and, with their permission, shared their contact information with the first author. The first author then contacted interested individuals, provided information about the study and obtained informed consent. Inclusion criteria were having experience of encountering ambulance staff and living in a stigmatized neighbourhood. After the interviews, participants were invited to refer additional individuals who met the inclusion criteria. This provided three additional recruits, and a total of 18 persons participated in the study (Table 1), which was considered sufficient as the participants' narratives collectively provided both depth and variation in lived experiences of the phenomenon. The number of individuals who declined is unknown due to recruitment via gatekeepers and snowball sampling, and reasons were not systematically collected but some expressed concerns about sharing experiences for fear of affecting future care.

**TABLE 1:** Characteristics of participants (*n* = 18).

Characteristics	
Age, median (min‐max)	53.5 (19–67)
Female/Male, *n*	5/13
Born outside Sweden, *n*	18^a^
Time living in Sweden (years), median (min‐max)	14 (8–37)
Employment (yes/no/other^b^), *n*	9/1/8
Level of education, *n*	
Below elementary school	1
Elementary school	1
High school	6
University	6
Vocational training	4

^a^ Iraq, Jordan, Afghanistan, Syria, Somalia, Kuwait, Lebanon and Ethiopia.

^b^ Long‐term sick leave, student, retired.

### 2.3 Data Collection

Data were collected between June and October 2023 through narrative interviews, all of which were conducted solely by the first author (female), who had no prior relationship with any of the participants. In total, 13 individual interviews and two group interviews (with two and three participants) were conducted, seven with interpreter support (four with a professional interpreter and three with a relative). All interviews were conducted face‐to‐face except for one telephone interview. The interviews began with an open question inviting participants to describe their lived experiences of encountering ambulance staff, followed by narrative‐based questions. Brief notes were taken during interviews to support follow‐up questions and facilitate transcription. The interview guide was pilot‐tested and retained without revision, as the opening question elicited rich narratives, and the pilot interview was therefore included in the analysis. An openness to later revisions (e.g., due to interpreter use) was adopted but deemed unnecessary. The interviews were audio‐recorded, had a median (min‐max) duration of 31.5 (22–54) minutes and were transcribed verbatim by the first author.

### 2.4 Data Analysis

All data were analyzed according to the phenomenological hermeneutical approach of Lindseth and Norberg [10]. In line with Ricoeur's hermeneutics [12], the text was interpreted as a whole, where meaning is understood to emerge from the text itself rather than from interactions, impressions, or distinctions related to the form of data collection [10]. First, a *naive understanding* was developed through repeated readings of the transcripts to grasp the overall meaning of encountering ambulance staff in stigmatized neighbourhoods. This understanding was then formulated using a metaphor to capture emotional and existential dimensions [10]. All authors read the transcripts and jointly discussed the first author's naive understanding. The metaphor used to express this understanding was not immediately clear to all authors, and differing interpretations were raised, allowing different pre‐understandings to be reflected upon and critically examined. The first and last author then revisited the transcripts and refined the naive understanding, which was subsequently reviewed and discussed by all authors until consensus was reached. A *structural analysis* was then conducted, in which the first author identified and condensed meaning units and organized them into subthemes in dialogue with the last author. Throughout this phase, interpretations were continuously reflected upon and discussed among the authors to ensure a critical and reflexive stance. These themes were then reviewed and refined by all authors until consensus was reached (see Table 2 for examples). Finally, a *comprehensive understanding* was developed by integrating the naive understanding and structural analysis. This synthesis was read, discussed and jointly refined by all authors, with pre‐understandings critically reflected upon throughout the process, allowing the interview material and relevant theories to inform one another. As the authors' differing pre‐understandings led to somewhat different interpretations of the material, three theories with distinct perspectives were drawn upon to integrate rather than exclude these understandings: caring and uncaring encounters [15], the model of person‐centeredness [16] and the theory of minority stress [17].

**TABLE 2:** Examples from the structural analysis.

Meaning units	Condensation	Subtheme	Theme	Main theme
Because I remember, time and time again, I said to them: he was feeling very, very bad! I really tried to show them that he was feeling very bad and that he really needed to go in! [to the emergency department]/…/ It felt like I really needed to show them that he really needed help…I really tried to show them how things had been so that they understood that it was bad enough that they should take him with them.	Having to show suffering to get the help needed.	Being in a fight for acceptance	Facing a double burden of coping and proving the suffering	Striving to be of equal value
They[ambulance staff] said you can come to the emergency room yourself and wait there/…/So, they went by themselves with those who have been in the crash/…/It was hard! It wasn't good, my brother was little, we wanted to be there to see him, so he didn't feel even worse. But we weren't allowed to participate.	Having to accept the ambulance staff's decisions even though they were not in compliance with own needs.	Being inferior to another	Being lost in the shadow of another's power	
I wasn't important for them [ambulance staff] and they couldn't answer me when it came to these questions that I had. They didn't answer, but when it came to the removal, they placed the responsibility on me [to handle the removal], even though it was in fact the responsibility of the police. I had questions and didn't get answers from them. Then you felt unimportant for them.	Not being of importance, with needs not acknowledged by the ambulance staff.	Being unseen in needs	Being lost in the shadow of another's power	

### 2.5 Ethical Considerations

All participants received both written and oral information about the study and were informed of their right to withdraw at any time, as well as of the researcher's background, including occupation, ethnocultural background and experience of living in stigmatized neighbourhoods. Written informed consent was then obtained by the first author. The participants could choose when, where and how the interview would be conducted, including the format (in person or by telephone), use of an interpreter, location, timing and whether the interview was individual or in a group. During the interviews, attention was paid to indications that participants wished to withdraw or change the subject. No participant wished to withdraw, but signals indicating a desire to change the subject were observed, for example, when a participant began talking about care in another context. Nevertheless, participants typically returned to the original subject and further elaborated on their narratives. After each interview, participants were offered time to discuss any emerging feelings, and contact information for follow‐up questions was provided. The interview transcripts were de‐identified, coded, password‐protected and stored on an encrypted university server.

## 3 Findings

### 3.1 Naive Understanding

Encountering ambulance staff when living in a stigmatized neighbourhood is to be exposed to their interpersonal actions in a situation overshadowed by fear; not only fear for one's health, but fear of being judged as less valuable due to background or place of residence. This interaction, where both the need for care and a sense of fear coexist, can be seen as a fragile rhythm that reflects the dynamic between the person and the ambulance staff. When the staff's empathy and skill guide the encounter, the rhythm becomes harmonious, allowing trust to grow and the person to feel seen and accepted. But the rhythm can also become disharmonious when one is doubted, degraded, or not believed and a fear of being abandoned grows. Attempts to smooth the rhythm by adapting to the staff's expectations may arise, or the encounter may turn into a struggle for recognition expressed through efforts to convince, explain, or display one's suffering. The longer this disharmony persists, the more one begins to question one's own understanding and right to receive care, accompanied by growing doubts about one's worth as an equal human being.

### 3.2 Structural Analysis

The structural analysis resulted in the main theme *Striving to be of equal value*, comprising three themes and six subthemes supported by citations (Table 3).

**TABLE 3:** Main theme, themes, subthemes and quotations.

Main theme	Theme	Subtheme	Quotation
Striving to be of equal value	Facing a double burden of coping and proving the suffering	Being inadequate and in need of assistance	‘I was really stressed because my dad has heart problems, and I kept thinking, I can't do anything. Then he said he was having a heart attack, and I thought, if something happens to his heart now, the rest of my life will be ruined. I was stressed because I had to keep an eye on my dad and talk to them at the same time, because they were asking questions. I don't know exactly how he feels. I can't speak for everything he feels. He might have described something, but I could end up explaining it the wrong way or missing something. It's different when someone speaks for another person. If my dad had been able to speak [the language] when he was feeling that bad and had talked to them himself, they would have understood how serious the pain really was. He needed emergency surgery, so it was really bad.’ (Participant 1)
		Being in a fight for acceptance	2 ‘It was enough that he said we were screaming and yelling and that he didn't understand anything. I said, what is it that you don't understand? He [the ambulance staff] said it was derogatory because it was from another culture. And I thought: what are you even thinking when you say that? Maybe to you it sounds like we're screaming and yelling. I don't know how it sounds to you. But when we were screaming, it was because we were scared. We weren't screaming at you. Why are you bringing up culture now, when this is an emergency? I told him, calmly, that he needed to be more careful with how he spoke. Then he talked to another health care worker on the phone, reporting to the emergency room, and said: I have a very difficult family, a difficult relative. They're yelling and screaming, and I don't understand anything. I said: what is it you don't understand? I can explain it, just don't talk nonsense. And then he reported: I'm warning you, a difficult relative is coming in. I told him: That doesn't belong here. That has nothing to do with this. I said: I'm sitting right here. You're talking about us like that while I'm sitting in the car. Think about what you're saying.’ (Participant 2)
Striving to be of equal value	Being lost in the shadow of another's power	Being inferior to another	3 ‘Every time I've called an ambulance, it's been different. Honestly, I don't even call an ambulance anymore, there's a risk in calling. It all depends on who shows up, and I don't think it should be like that. You need to separate your personal life from your work. When you're at work, you should focus on your job and leave everything else aside. You should have a certain professional approach. The first thing that goes through my mind [when the ambulance staff arrive] is: Is he racist or not?’ (Participant 12)
		Being unseen in needs	4 ‘The person having the seizure, he was shaking, and sometimes there was this sound, kind of strange… I understood it as if he was having trouble breathing. It was a serious situation! The reason we called [the ambulance service] was because we expected them to take over and help calm the situation down. We thought they would check his pulse or see if he was breathing normally, things like that, but that's not what happened. /…/When they arrived, he was still having the seizure. We were holding him because he's heavy, and we were scared he might roll over and not be able to breathe. I thought they would come in, assess the situation, and help. Instead, she stood there and asked: has he been drinking? /…/In the end, they took my son into the ambulance, but I had a really bad feeling. It was about my son, and about the language she [ambulance staff] used in that moment. I mean, the person was having an epileptic seizure so in what situation are those kinds of words appropriate? We had called her for help, as a professional.’ (Participant 13)
Striving to be of equal value	Being in a space of safety	Being worthy of care	5 ‘She [ambulance staff] sang to her and took care of her, and I felt safe, you know. Someone was really taking care of my daughter. She didn't just sit there and do her job, she did more than that! She joked with her, she sang to her, and yes, I felt happy and safe. My daughter cried a little, then laughed a little, and it just felt good. She took care of her the best she could! /…/She calmed her down, talked to her, and talked to me too! If I had been sitting there alone for an hour, worrying about my daughter, it would have been unbearable. But she talked to me, asked me about my work and things like that. I ended up talking a lot, and for a moment I forgot what was happening. That really helped. I needed someone to talk to as well, because I felt my daughter was getting worse and worse. I really needed to talk to someone.’ (Participant 3)
		Being in togetherness	6 ‘They [ambulance staff] went straight to him! They didn't look around or focus on the family or anything like that. They didn't ask who everyone was, which has happened several times before. They went straight to him, checked him, took his temperature and blood pressure. They got to work right away! I was there to help if they needed anything, and I really appreciated that. It felt professional. That's how it should be, once you arrive, you start the job you're there to do. He [patient] was confused and a bit unsure, so I said, I'll take care of this. I told him to bring his jacket. We helped get him ready, brought his pants and a shirt, and I took him out to the ambulance.’ (Participant 18)

### 3.3 Main Theme: Striving To Be of Equal Value

Encountering ambulance staff while living in a stigmatized neighbourhood is a struggle for equal value. The value is assessed based on the ambulance staff's approach, intentions and decisions that form their way of being. This is to be in a power dynamic, subordinated to the ambulance staff, where concern about being judged based on background involving ethnicity, culture, or neighbourhood may grow. To achieve relief from suffering, the ambulance staff's behaviour is observed in order to understand how to act to be believed and receive appropriate care. Whether needs are acknowledged and a person's value is validated depends on the ambulance staff's way of being. Being diminished due to background may emerge, where doubt about one's own worth as well as further suffering can grow. However, meanings of being seen as well as safe can also develop where one's value as an equal person is confirmed when needs are acknowledged and addressed.

### 3.4 Theme 1: Facing a Double Burden of Coping and Proving the Suffering

Facing a double burden entails managing suffering while striving to communicate needs despite language barriers or differing understandings, alongside evaluating the ambulance staff's approach to interpret whether those needs are acknowledged. It is a fight for validation where the severity of the situation must be proved despite a fear of being distrusted or dismissed due to one's own background involving ethnicity, culture, or neighbourhood.

#### 3.4.1 Subtheme: Being Inadequate and in Need of Assistance

To be inadequate entails being in a situation where demand exceeds ability. It is to be responsible for managing suffering while being afraid of threats to health and well‐being, without knowing what to do. To receive help, attempts are made to articulate needs while, at the same time, one struggles to understand them and how they should be expressed (Table 3, Quotation 1).

Encountering ambulance staff involves being a mediator of suffering, where attempts are made to overcome language barriers or different understandings of the situation. One's bodily and verbal expressions become ways of making suffering understandable. At the same time, attention is directed towards how words and expressions are interpreted by the ambulance staff and whether their actions respond to needs. This serves as an indicator of whether suffering and needs are correctly expressed, perceived and understood.

#### 3.4.2 Subtheme: Being in a Fight for Acceptance

Being in a fight for acceptance involves expressing suffering by explaining or convincing the ambulance staff that care needs exist and that one should not be abandoned. One is in fear that needs will not be understood as genuine care needs but instead will be interpreted by the ambulance staff in relation to background, such as ethnicity, culture, or neighbourhood. When needs are perceived as dismissed due to background, the encounter is marked by distrust or neglect (Table 3, Quotation 2). Then, the fight is not only for one's needs to be acknowledged but also for one's own value as an equally unique person. Being distrusted and neglected transforms the encounter from one of expected relief into one of further suffering in which help is absent. Suffering is managed alone, while other help options are considered to avoid future encounters with ambulance staff.

### 3.5 Theme 2: Being Lost in the Shadow of Another's Power

Being lost in the shadow of another's power is to be overshadowed by the ambulance staff's authority. Relief emerges when needs are seen but self‐doubt about the justification of care grows when the needs are not acknowledged. The encounter then becomes one of adaptation, in which one adjusts one's behaviour and explanations in order to receive any care at all. Doubt arises as to whether the ambulance staff are authentic and reliable in their intentions; at the same time, gratitude is experienced for the help that is attempted.

#### 3.5.1 Subtheme: Being Inferior to Another

Being inferior to another is to be dependent on the one arriving to help, while being uncertain if help will be delivered. Hence, fear of being judged due to one's ethnicity, culture, or neighbourhood exists and the ambulance staff's approach is carefully evaluated in an effort to understand their intentions (Table 3, Quotation 3).

The ambulance staff's perceived intentions guide what to expect in the encounter. Relief emerges when the ambulance staff's approach is perceived as acknowledging needs, and their intentions are believed to reflect a genuine willingness to provide care. When doubt exists about the willingness, the patient's own role is adapted according to what is considered necessary to receive the required care. Adaptation entails being submissive towards the ambulance staff's decisions or arrangements and following their instructions. When the ambulance staff's intentions are not perceived as genuinely caring, one submits to their decisions and arrangements, even though needs remain unmet, in order to receive any form of care.

#### 3.5.2 Subtheme: Being Unseen in Needs

Being unseen is to be unacknowledged or dismissed as regards one's unique needs, where the ambulance staff's perspective dictates care. Encountering ambulance staff entails receiving care solely based on another's understanding, but this care may be inadequate or entirely absent (Table 3, Quotation 4).

When needs are not acknowledged or are dismissed, doubt about one's own experiences emerges. When one feels diminished or suppressed based on ethnicity, culture, or neighbourhood, the encounter is judged to be of lesser value to the ambulance staff. Hence, doubt about one's own worth grows and the encounter becomes characterized by withdrawal and compliance with another's wishes. However, gratitude for the attempt to provide help remains even though one's needs are not fulfilled, while at the same time, distrust about the authenticity of the ambulance staff grows.

### 3.6 Theme 3: Being in a Space of Safety

Being in a space of safety means to be relieved of the responsibility for managing the suffering by oneself. Hence, the ambulance staff's approach shows that they care and acknowledge needs. The encounter is supported based on a mutual collaboration that confirms one's own value as an equal human being.

#### 3.6.1 Subtheme: Being Worthy of Care

Being worthy of care means being seen as a person with a legitimate right to care. Being recognized as worthy affirms one's own value as an equal human being. Then one is seen through the lens of uniqueness, presenting needs, rather than being judged by prejudice. To be deemed worthy is to have those needs, extending beyond mere medical requirements, acknowledged, which makes the suffering more bearable (Table 3, Quotation 5).

The responsibility for managing the suffering is transferred to someone who accepts it and who is trying to provide alleviation. One's own experiences and perspectives become the primary focus of the ambulance staff in the encounter, where wishes as well as conditions are listened to. Encountering ambulance staff then means one is cared for, as someone else supports the management of suffering based on one's unique needs.

#### 3.6.2 Subtheme: Being in Togetherness

Being in togetherness is to be in a collaboration where both encountering parties value each other in a mutuality. Decisions and arrangements are made together and all experiences are acknowledged. It means being safe, where experiences and needs can be shared without being judged by background (Table 3, Quotation 6).

The encounter is essential in the formation of care and inclusion, where one's contribution is recognized. Care is developed along with a shared goal formed in the moment when working side by side with the ambulance staff to alleviate suffering.

### 3.7 Comprehensive Understanding

Encountering ambulance staff while living in a stigmatized neighbourhood entails striving for equal value in an interaction that extends beyond the technical aspects of care [15]. It means being in a situation overshadowed by fear, not only regarding one's own or a relative's health, but also fear of being judged due to ethnicity, culture, or place of residence. The encounter involves being in a power relation shaped both by the immediate encounter and by broader structures of injustice [17]. These social structures form a wall, obstructing the possibility of a shared relationship where power can be more evenly distributed [15, 17]. One is lost in the shadow of another's power, dependent on whether the ambulance staff recognise one's personhood [15, 16].

When personhood is not acknowledged, questioning of one's own understanding and the legitimacy of care emerges. One may subordinate oneself to avoid provoking those in power, fearing being left without help [17]. Doubts about self‐worth may arise, fueled by feelings of being generalized or categorized according to societal structures, reinforcing suffering [15, 17]. Being disbelieved or degraded intensifies the fear of abandonment, and attempts to resist subordination emerge through striving to be acknowledged as a person [17]. Thus, encountering ambulance staff while living in a stigmatized neighbourhood entails a double burden of coping and proving the suffering where one experiences stress not only related to the need for care but to the need to be acknowledged as an equal human [15, 17].

When the ambulance staff's way of being is characterized by empathy and professional skill, personhood is affirmed and unique needs acknowledged. Trust grows, enabling a relational bridge to form [15]. The encounter becomes a partnership in which care is co‐created, making suffering more manageable [16]. Within this togetherness, the capacity to cope increases as suffering is shared, and one is experienced as worthy of a genuine relationship, strengthening one's sense of human value [15, 17]. The encounter then becomes a space of safety where one is being cared for, seen and accepted as a person within the staff's relational presence [15]. One becomes essential in shaping the care, participating as an equal where both parties contribute with their respective expertise [16].

## 4 Discussion

The findings indicate that encountering ambulance staff while living in a stigmatized neighbourhood is shaped by power imbalances. This can be further understood through the lens of the theory of street‐level bureaucracy by Lipsky [18], where ambulance staff, as frontline workers, exercise discretion in encounters that are shaped by non‐voluntary situations. The non‐voluntary nature of these encounters stems from patients' and relatives' dependence on urgent care and their limited alternatives, creating an inherent power asymmetry that shapes how they adapt [19, 20]. The findings reveal a fear of being judged based on ethnicity, culture, or place of residence influencing whether they feel able to express needs or feel compelled to comply. This risk of being stereotyped in health care encounters with ambulance staff is supported by previous research [8, 9]. Understood from the perspective of street‐level bureaucracy [18], frontline workers such as ambulance staff engage in processes of categorization and differentiation, which may be influenced by broader societal stereotypes, as a way to manage complex and time–pressure situations [18]. Taken together, this suggests that power in AS encounters is not only exercised at the frontline but is structurally produced, where discretionary practices may be influenced by wider societal inequalities.

In contrast to these challenges, the findings also highlight how encounters characterized by empathy and professional competence can transform the care experience. When ambulance staff demonstrate sensitivity, presence and clinical skills, patients and relatives experience being seen as persons rather than as a category based on ethnicity, culture, or place of residence. From a caring theoretical perspective, this can be understood as openness enabling genuine caring to develop despite surrounding influences [21]. In this light, the findings point to the potential within frontline discretion from the perspective of street‐level bureaucracy [18], where ambulance staff can act as agents of change in their everyday practice. By fostering openness through conscious reflection on their discretionary actions, ambulance staff may challenge taken‐for‐granted routines and contribute to more equitable care [9, 22]. However, improving such practices also requires organizational support that enables time, discretion and conditions for encounters that are more responsive to different needs informed by the clients, who are typically underrepresented in the development of practice and policy [18]. In this context, incorporating patients' and relatives' perspectives may help counterbalance existing power asymmetries and enhance responsiveness in care and could be an important focus for future research.

### 4.1 Methodological Considerations

Given the aim to illuminate meanings of a phenomenon, a phenomenological hermeneutic approach was adopted, as it strengthens interpretative depth. However, other approaches might have generated more descriptive or contextual insights, highlighting different aspects of health care encounters in stigmatized neighbourhoods. Within the phenomenological hermeneutical approach, credibility is grounded in a rigorous interpretative process, where understanding develops through a dialectical movement between the whole and its parts and where preunderstanding is continuously reflected upon and revised in dialogue with the text [23]. The researchers' diverse preunderstandings contributed to a nuanced interpretation while also shaping the findings as one possible understanding of the phenomenon. These may also have influenced interview situations by shaping follow‐up questions but may likewise have facilitated deeper understanding by enabling sensitivity to participants' meanings. While the research team had experience of AS encounters, and one author (S.B.) has a diverse ethnic and cultural background and experience of living in stigmatized neighbourhoods (but no migration‐related experience), other preunderstandings may have led to different interpretations.

Within phenomenological hermeneutics, rich and varied narrative data are essential, as they provide the foundation for a nuanced interpretation of lived experience [23]. In this study, the potential for variation may be limited, as participants were all foreign‐born, predominantly men and recruited from a single stigmatized neighbourhood. The use of gatekeepers may also have constrained variation by favouring more socially integrated individuals, potentially excluding more isolated residents. To increase variation and reduce barriers to participation, interpreters were used, which can risk limiting depth. However, both professional and relative interpreters appeared to facilitate rather than constrain the interviews by enabling participants to express their experiences in their own language. When relatives acted as interpreters, existing trust and contextual knowledge seemed to enrich the narratives. Similarly, offering group interviews appeared to enhance comfort and openness. However, some interviews were relatively short but still rich in data, and although repeated interviews might have provided greater depth, these were not conducted due to the complexity of recruitment.

## 5 Conclusion

Encounters with ambulance staff carried meanings of fear of being judged and of dependence, while ambulance staff's interpersonal actions appeared important in either reinforcing these meanings or creating possibilities for recognition, participation and acknowledgment as a person of equal value.

## Author Contributions

**Sara Björklund:** interview, data curation, formal analysis, writing and original draft; **Peter Hagell:** project administration, formal analysis, review and editing, funding acquisition. **Petra Lilja Hagell:** formal analysis, review and editing. **Mats Holmberg:** supervision, methodology, formal analysis, review and editing. All authors have prior experience conducting qualitative interview studies and working with interpreters in professional settings, and author M.H. has extensive experience with phenomenological hermeneutic research.

## Funding

This study was supported by Region Blekinge and Jane and Dan Olssons Stiftelse.

## Ethics Statement

The study was approved by the Swedish Ethical Review Authority (No. 2021–06744‐01; No. 2023–02080‐02) and conducted in accordance with the Declaration of Helsinki. Written consent was obtained from all participants before the interviews.

## Conflicts of Interest

The authors declare no conflicts of interest.

## Data Availability

The data that support the findings of this study are available on request from the corresponding author. The data are not publicly available due to privacy or ethical restrictions.
